# Author Correction: Finite-key analysis for twin-field quantum key distribution with composable security

**DOI:** 10.1038/s41598-020-69089-6

**Published:** 2020-07-16

**Authors:** Hua-Lei Yin, Zeng-Bing Chen

**Affiliations:** National Laboratory of Solid State Microstructures and School of Physics, Nanjing University, Nanjing, 210093 China

Correction to:* Scientific Reports* 10.1038/s41598-019-53435-4, published online 19 November 2019

This Article contains typographical errors in the Introduction.

“Recently, we became aware that the single-photon based protocol and its key rate formula, Eq. (3) presented in Ref. [21] are actually same with the earlier protocol, sending-or-not-sending protocol proposed by Wang et al. We thank authors of Ref. [22] for pointing out this.”

should read:

“Recently, we became aware that the single-photon based protocol and its key rate formula, Eq. (3) presented in Ref. [22] are actually same with the earlier protocol [21], sending-or-not-sending protocol proposed by Wang et al. We thank authors of Ref. [21] for pointing out this.”

