# Supplementary material for: Finite-key analysis for twin-field quantum key distribution with composable security
Source: Sci Rep. 2019 Nov 19;9:17113. doi: 10.1038/s41598-019-53435-4 (PMC6863895; doi:10.1038/s41598-019-53435-4)
Supplement: Supplementary file 1 — Supplementary information [file 41598_2019_53435_MOESM1_ESM.pdf]

Supplemental Material for "Finite-Key Analysis for Twin-Field Quantum Key Distribution with Composable Security"

Hua-Lei Yin[1,*] and Zeng-Bing Chen[1,†]

[1]*National Laboratory of Solid State Microstructures and School of Physics, Nanjing University, Nanjing 210093, China*

Supplementary Note 1: Random sampling without replacement.

Here, we present the proof for the lemma of random sampling without replacement in the Methods of the main text. The new tail inequality of random sampling without replacement is the tightest due to avoiding any inequality scaling.

Lemma 1. Tight tail inequality of random sampling without replacement.

Let $\mathcal{X}_{n+k} := \{x_1, x_2, \cdots, x_{n+k}\}$ be a string of binary bits with $n+k$ size, in which the number of bit value 1 is unknown. Let $\mathcal{X}_k$ be a random sample (without replacement) bit string with k size from $\mathcal{X}_{n+k}$. Let λ_k be the probability of bit value 1 observed in $\mathcal{X}_k$. Let $\mathcal{X}_n$ be the remaining bit string, where the probability of bit value 1 observed in $\mathcal{X}_n$ is λ_n. Then let $C_i^j = i!/[j!(i-j)!]$ be the binomial coefficient. For any $\epsilon > 0$, we have the upper tail

$$\Pr[\lambda_n \geq \lambda_k + \gamma(n, k, \lambda_k, \epsilon)] \leq \epsilon, \tag{1}$$

where $\gamma(a, b, c, d)$ is the positive root of the following equation

$$\ln C_b^{bc} + \ln C_a^{ac+a\gamma(a,b,c,d)} - \ln C_{a+b}^{(a+b)c+a\gamma(a,b,c,d)} - \ln d = 0. \tag{2}$$

For any $\hat{\epsilon} > 0$, we have the lower tail

$$\Pr[\lambda_n \leq \lambda_k - \hat{\gamma}(n, k, \lambda_k, \hat{\epsilon})] \leq \hat{\epsilon}, \tag{3}$$

where $\hat{\gamma}(a, b, c, d)$ is the positive root of the following equation

$$\ln C_b^{bc} + \ln C_a^{ac-a\hat{\gamma}(a,b,c,d)} - \ln C_{a+b}^{(a+b)c-a\hat{\gamma}(a,b,c,d)} - \ln d = 0. \tag{4}$$

If one does not find the positive root $\hat{\gamma}(a, b, c, d)$, we let $\lambda_n = 0$.

Proof.

First, we prove the inequality of the upper tail. Let $X = n\lambda_n + k\lambda_k$, we have

$$\begin{aligned}
\Pr[\lambda_n \geq \lambda_k + \gamma] &= \Pr[X \geq (n+k)\lambda_k + n\gamma, k\lambda_k] \\
&= \sum_{X=(n+k)\lambda_k+n\gamma}^{n+k\lambda_k} \Pr[X, k\lambda_k] \\
&= \sum_{X=(n+k)\lambda_k+n\gamma}^{n+k\lambda_k} \Pr[k\lambda_k|X]\Pr[X] \\
&= \sum_{X=(n+k)\lambda_k+n\gamma}^{n+k\lambda_k} \frac{C_k^{k\lambda_k} C_n^{X-k\lambda_k}}{C_{n+k}^X}\Pr[X] \\
&\leq \frac{C_k^{k\lambda_k} C_n^{n\lambda_k+n\gamma}}{C_{n+k}^{(n+k)\lambda_k+n\gamma}},
\end{aligned} \tag{5}$$

*Electronic address: hlyin@nju.edu.cn

†Electronic address: zbchen@nju.edu.cn

where we use the fact that the conditional probability $\Pr[k\lambda_k|X] = C_k^{k\lambda_k} C_n^{X-k\lambda_k}/C_{n+k}^X$ is the hypergeometric distribution function and is a monotonic decreasing function of X when $X \geq (n+k)\lambda_k$. By using Eq. (2), we find

$$\frac{C_k^{k\lambda_k} C_n^{n\lambda_k+n\gamma(n,k,\lambda_k,\epsilon)}}{C_{n+k}^{(n+k)\lambda_k+n\gamma(n,k,\lambda_k,\epsilon)}} = \epsilon. \tag{6}$$

Thereby, we have proved the upper tail $\Pr[\lambda_n \geq \lambda_k + \gamma(n,k,\lambda_k,\epsilon)] \leq \epsilon$.

Now, we prove the inequality of the lower tail. We consider the case of $\lambda_k \geq \hat{\gamma}(n,k,\lambda_k,\hat{\epsilon}) \geq 0$. Let $\hat{X} = n\lambda_n + k\lambda_k$, we have

$$\begin{aligned}
\Pr[\lambda_n \leq \lambda_k - \hat{\gamma}] &= \Pr[\hat{X} \leq (n+k)\lambda_k - n\hat{\gamma}, k\lambda_k] \\
&= \sum_{\hat{X}=k\lambda_k}^{(n+k)\lambda_k-n\hat{\gamma}} \Pr[\hat{X}, k\lambda_k] \\
&= \sum_{\hat{X}=k\lambda_k}^{(n+k)\lambda_k-n\hat{\gamma}} \Pr[k\lambda_k|\hat{X}]\Pr[\hat{X}] \\
&= \sum_{\hat{X}=k\lambda_k}^{(n+k)\lambda_k-n\hat{\gamma}} \frac{C_k^{k\lambda_k} C_n^{\hat{X}-k\lambda_k}}{C_{n+k}^{\hat{X}}} \Pr[\hat{X}] \\
&\leq \frac{C_k^{k\lambda_k} C_n^{n\lambda_k-n\hat{\gamma}}}{C_{n+k}^{(n+k)\lambda_k-n\hat{\gamma}}},
\end{aligned} \tag{7}$$

where we use the fact that the conditional probability $\Pr[k\lambda_k|\hat{X}] = C_k^{k\lambda_k} C_n^{\hat{X}-k\lambda_k}/C_{n+k}^{\hat{X}}$ is the hypergeometric distribution function and is a monotonic increasing function of $\hat{X}$ when $\hat{X} \leq (n+k)\lambda_k$. By using Eq. (4), we find

$$\frac{C_k^{k\lambda_k} C_n^{n\lambda_k-n\hat{\gamma}(n,k,\lambda_k,\hat{\epsilon})}}{C_{n+k}^{(n+k)\lambda_k-n\hat{\gamma}(n,k,\lambda_k,\hat{\epsilon})}} = \hat{\epsilon}. \tag{8}$$

Thereby, we have proved the lower tail $\Pr[\lambda_n \leq \lambda_k - \hat{\gamma}(n,k,\lambda_k,\hat{\epsilon})] \leq \hat{\epsilon}$.

Supplementary Note 2: The multiplicative Chernoff bound and its variant.

Here, we give the proof for the Lemma of the multiplicative Chernoff bound and its variant shown in the Methods of the main text. First, we prove that the multiplicative Chernoff bound is almost the tightest. The multiplicative Chernoff bound is exploited to estimate the observed value, given the expected value. Second, we propose a variant of the multiplicative Chernoff bound as tight as possible which is used to bound the expected value, given the observed value.

Lemma 2. Tight multiplicative Chernoff bound.

Let $X_1, X_2 ..., X_N$ be a set of independent Bernoulli random variables that satisfy $\Pr(X_i = 1) = p_i$ (not necessarily equal), and let $X := \sum_{i=1}^N X_i$. The expected value of X is denoted as $\mu_x := E[X] = \sum_{i=1}^N p_i$. Then, let $g(x,y) = \left[\frac{e^y}{(1+y)^{1+y}}\right]^x$, for any $\delta > 0$, we have the upper tail

$$\Pr[X \geq (1+\delta)\mu_x] < g(\mu_x, \delta) = \epsilon, \tag{9}$$

where δ is the positive root of the following equation

$$\mu_x[\delta - (1+\delta)\ln(1+\delta)] - \ln\epsilon = 0. \tag{10}$$

For any $0 < \hat{\delta} \leq 1$, we have the lower tail

$$\Pr[X \leq (1-\hat{\delta})\mu_x] < g(\mu_x, -\hat{\delta}) = \hat{\epsilon}, \tag{11}$$

where $\hat{\delta}$ is the positive root of the following equation

$$\mu_x[\hat{\delta} + (1-\hat{\delta})\ln(1-\hat{\delta})] + \ln\hat{\epsilon} = 0. \tag{12}$$

Proof.

First, we prove the first inequality of upper tail. For $t > 0$, we can have an equivalent inequality,

$$\Pr[X \geq (1+\delta)\mu_x] = \Pr[e^{tX} \geq e^{t(1+\delta)\mu_x}]. \tag{13}$$

By exploiting the Markov inequality, the above inequality can be given by

$$\Pr[X \geq (1+\delta)\mu_x] = \Pr[e^{tX} \geq e^{t(1+\delta)\mu_x}] \leq \frac{E[e^{tX}]}{e^{t(1+\delta)\mu_x}}. \tag{14}$$

Since $X = \Sigma_{i=1}^{N} X_i$, we have $E[e^{tX}] = \Pi_{i=1}^{N} E[e^{tX_i}]$. The independent Bernoulli random variables satisfy $\Pr(X_i = 1) = p_i$. The expected value is $E[e^{tX_i}] = 1 + p_i(e^t - 1) < e^{p_i(e^t-1)}$, where we use the fact that $e^y > (1+y)$ for $y > 0$. Thereby, we have the inequality

$$E[e^{tX}] = \prod_{i=1}^{N} E[e^{tX_i}] < \prod_{i=1}^{N} e^{p_i(e^t-1)} = e^{\sum_{i=1}^{N} p_i(e^t-1)} = e^{(e^t-1)\mu_x}. \tag{15}$$

Substituting Eq. (15) back into Eq. (14), the final inequality can be bounded by

$$\Pr[X \geq (1+\delta)\mu_x] < \frac{e^{(e^t-1)\mu_x}}{e^{t(1+\delta)\mu_x}} = \left[\frac{e^{\delta}}{(1+\delta)^{1+\delta}}\right]^{\mu_x}, \tag{16}$$

where we assume $t = \ln(1+\delta)$ to **make the bound as tight as possible**. By using Eq.(10), we have

$$\Pr[X \geq (1+\delta)\mu_x] <= \left[\frac{e^{\delta}}{(1+\delta)^{1+\delta}}\right]^{\mu_x} = g(\mu_x, \delta) = \epsilon. \tag{17}$$

Now, we prove the second inequality of lower tail by using the similar method. For $t > 0$, we have an equivalent inequality as follows,

$$\Pr[X \leq (1-\hat{\delta})\mu_x] = \Pr[e^{-tX} \geq e^{-t(1-\hat{\delta})\mu_x}]. \tag{18}$$

The above inequality can be bounded by the Markov inequality,

$$\Pr[X \leq (1-\hat{\delta})\mu_x] = \Pr[e^{-tX} \geq e^{-t(1-\hat{\delta})\mu_x}] \leq \frac{E[e^{-tX}]}{e^{-t(1-\hat{\delta})\mu_x}}. \tag{19}$$

Obviously, $E[e^{-tX}] = \Pi_{i=1}^{N} E[e^{-tX_i}]$ because $X = \Sigma_{i=1}^{N} X_i$. The expected value is $E[e^{-tX_i}] = 1 + p_i(e^{-t} - 1) < e^{p_i(e^{-t}-1)}$ since the independent Bernoulli random variables satisfy $\Pr(X_i = 1) = p_i$, where we use the fact that $e^y > (1+y)$ for $-1 < y < 0$. Thereby, the expected value $E[e^{-tX}]$ can be written as

$$E[e^{-tX}] = \prod_{i=1}^{N} E[e^{-tX_i}] < \prod_{i=1}^{N} e^{p_i(e^{-t}-1)} = e^{\sum_{i=1}^{N} p_i(e^{-t}-1)} = e^{(e^{-t}-1)\mu_x}. \tag{20}$$

Substituting Eq. (20) back into Eq. (19), the final inequality can be bounded by

$$\Pr[X \leq (1-\hat{\delta})\mu_x] < \frac{e^{(e^{-t}-1)\mu_x}}{e^{-t(1-\hat{\delta})\mu_x}} = \left[\frac{e^{-\hat{\delta}}}{(1-\hat{\delta})^{1-\hat{\delta}}}\right]^{\mu_x}, \tag{21}$$

where we assume that $t = -\ln(1-\hat{\delta})$ to **make the bound as tight as possible**. By using Eq.(12), we have

$$\Pr[X \leq (1-\hat{\delta})\mu_x] < \left[\frac{e^{-\hat{\delta}}}{(1-\hat{\delta})^{1-\hat{\delta}}}\right]^{\mu_x} = g(\mu_x, -\hat{\delta}) = \hat{\epsilon}, \tag{22}$$

Note that the above proof of the multiplicative Chernoff bound exploits the expected value μ_x, which means that **this bound requires the knowledge of μ_x.**

Lemma 3. A variant of the tight multiplicative Chernoff bound.

Let $X_1, X_2..., X_N$ be a set of independent Bernoulli random variables that satisfy $\Pr(X_i = 1) = p_i$ (not necessarily equal), and let $X := \sum_{i=1}^{N} X_i$. The expected value of X is denoted as $\mu_x := E[X] = \sum_{i=1}^{N} p_i$. An observed outcome of X is represented as x for a given trial (note that, we have $x \geq 0$, $\mu_x \geq 0$ and μ_x is unknown). For any $\epsilon > 0$, we have that μ_x satisfies

$$\mu_x \geq \underline{\mu_x} = \max\{0, x - \Delta(x, \epsilon)\}, \tag{23}$$

with failure probability ϵ, where $\underline{\mu_x}$ is the lower bound of μ_x and $\Delta(z, y)$ is the positive root of the following equation

$$\Delta(z, y) - [z + \Delta(z, y)] \ln \frac{z + \Delta(z, y)}{z} - \ln y = 0. \tag{24}$$

For any $\hat{\epsilon} > 0$, we have that μ_x satisfies

$$\mu_x \leq \overline{\mu_x} = x + \hat{\Delta}(x, \hat{\epsilon}), \tag{25}$$

with failure probability $\hat{\epsilon}$, where $\overline{\mu_x}$ is upper bound of μ_x and $\hat{\Delta}(z, y)$ is the positive root of the following equation

$$\hat{\Delta}(z, y) + z \ln \frac{z}{z + \hat{\Delta}(z, y)} + \ln y = 0. \tag{26}$$

Proof.

Here, we first prove the case of Eq. (23). Obviously, $\underline{\mu_x} \equiv 0$ if $x \leq \Delta(x, \epsilon)$, otherwise $\underline{\mu_x} = x - \Delta(x, \epsilon)$. We consider the case of $x > \Delta(x, \epsilon)$. We have $x > \underline{\mu_x}$ due to $\Delta(x, \epsilon) > 0$. The root $\Delta(z, y)$ of Eq. (24) is a monotonic increasing function of z given fixed y. The probability can be written as

$$\Pr[X \geq \mu_x + \Delta(X, \epsilon)] < \Pr[X > \underline{\mu_x} + \Delta(\underline{\mu_x}, \epsilon)], \tag{27}$$

where we exploit the fact that the observed outcome x of X for a given trial satisfies $x \geq \underline{\mu_x}$, $\mu_x \geq \underline{\mu_x}$ and $\Delta(z, y)$ is a monotonic increasing function of z given fixed y. By using the upper tail of the multiplicative Chernoff bound of **Lemma 2**, we have

$$\Pr[X \geq \underline{\mu_x} + \Delta(\underline{\mu_x}, \epsilon)] < \frac{e^{\Delta(\underline{\mu_x}, \epsilon)}}{[1 + \Delta(\underline{\mu_x}, \epsilon)/\underline{\mu_x}]^{\underline{\mu_x} + \Delta(\underline{\mu_x}, \epsilon)}}. \tag{28}$$

By using Eq. (24), we find that

$$\frac{e^{\Delta(\underline{\mu_x}, \epsilon)}}{[1 + \Delta(\underline{\mu_x}, \epsilon)/\underline{\mu_x}]^{\underline{\mu_x} + \Delta(\underline{\mu_x}, \epsilon)}} = \epsilon. \tag{29}$$

Therefore, we have the inequality

$$\Pr[X \geq \mu_x + \Delta(X, \epsilon)] < \Pr[X > \underline{\mu_x} + \Delta(\underline{\mu_x}, \epsilon)] = \epsilon, \tag{30}$$

which means that the probability of the observed outcome x of X for a given trial satisfying $x \geq \mu_x + \Delta(x, \epsilon)$ is less than ϵ. Combining the results above, we show that $\mu_x \geq \underline{\mu_x} = \max\{0, x - \Delta(x, \epsilon)\}$ with the failure probability at most ϵ.

Now, we prove the case of Eq. (25). Obviously, the root $\hat{\Delta}(z, y)$ of Eq. (26) is also a monotonic increasing function of z given fixed y. The probability can be written as

$$\Pr[X \leq \mu_x - \hat{\Delta}(X, \hat{\epsilon})] < \Pr[X < \overline{\mu_x}] = \Pr[X < \overline{\mu_x} + \hat{\Delta}(\overline{\mu_x}, \hat{\epsilon}) - \hat{\Delta}(\overline{\mu_x}, \hat{\epsilon})], \tag{31}$$

where we exploit the fact that the observed outcome x of X for a given trial satisfies $\hat{\Delta}(x, \hat{\epsilon}) > 0$ and $\mu_x \leq \overline{\mu_x}$. By using the lower tail of the multiplicative Chernoff bound of **Lemma 2**, we have

$$\Pr[X < \overline{\mu_x} + \hat{\Delta}(\overline{\mu_x}, \hat{\epsilon}) - \hat{\Delta}(\overline{\mu_x}, \hat{\epsilon})] < \frac{e^{-\hat{\Delta}(\overline{\mu_x}, \hat{\epsilon})}}{\left\{1 - \hat{\Delta}(\overline{\mu_x}, \hat{\epsilon})/[\overline{\mu_x} + \hat{\Delta}(\overline{\mu_x}, \hat{\epsilon})]\right\}^{\overline{\mu_x}}}. \tag{32}$$

By exploiting Eq. (26), we can find

$$\frac{e^{-\hat{\Delta}(\overline{\mu_x},\hat{\epsilon})}}{\left\{1-\hat{\Delta}(\overline{\mu_x},\hat{\epsilon})/\left[\overline{\mu_x}+\hat{\Delta}(\overline{\mu_x},\hat{\epsilon})\right]\right\}^{\overline{\mu_x}}}=\hat{\epsilon}. \tag{33}$$

Therefore, we have the inequality

$$\Pr[X\le\mu_x-\hat{\Delta}(X,\hat{\epsilon})]<\Pr[X<\overline{\mu_x}+\hat{\Delta}(\overline{\mu_x},\hat{\epsilon})-\hat{\Delta}(\overline{\mu_x},\hat{\epsilon})]=\hat{\epsilon}, \tag{34}$$

which means that the probability of the observed outcome x of X for a given trial satisfying $x\le\mu_x-\hat{\Delta}(x,\hat{\epsilon})$ is less than $\hat{\epsilon}$. Combining the results above, we show that $\mu_x\le\overline{\mu_x}=x+\hat{\Delta}(x,\hat{\epsilon})$ with the failure probability at most $\hat{\epsilon}$.

Note that the above proof of the variant of the tight multiplicative Chernoff bound does not exploit the expected value μ_x, which means that **this bound does not require the knowledge of μ_x**.

Supplementary Note 3: Comparing with previous methods of statistical fluctuation.

In this section, we will compare the statistical fluctuation analysis methods proposed in Notes 1 and 2 with previous works. First, we consider the statistical fluctuation of expected value, given the observed value. Here, we will introduce the rigorous variant of the Chernoff bound method proposed in [1] and the not-sufficiently-rigorous Gaussian analysis with the central limit theorem.

Lemma 4. A variant of the multiplicative Chernoff bound in [1].

Let $X_1, X_2, \ldots, X_N$, be a set of independent Bernoulli random variables that satisfy $\Pr(X_i=1)=p_i$ (not necessarily equal), and let $X=\sum_{i=1}^N X_i$ and $\mu_x=E[X]=\sum_{i=1}^N p_i$, where $E[\cdot]$ denotes the mean value. Let x be the observed outcome of X for a given trial (i.e., $x\in\mathbb{N}^+$) and $\mu_{\mathrm{L}}=x-\sqrt{N/2\ln(1/\epsilon)}$ for certain $\epsilon>0$. Then, we have that x satisfies

$$x=\mu_x+\delta, \tag{35}$$

except for error probability γ, where the parameter $\delta\in[-\Delta,\hat{\Delta}]$. Let $test_1$, $test_2$ and $test_3$ denote, respectively, the following three conditions: $\mu_{\mathrm{L}}\ge\frac{32}{9}\ln(2\varepsilon^{-1})$, $\mu_{\mathrm{L}}>3\ln(\hat{\varepsilon}^{-1})$ and $\mu_{\mathrm{L}}>\left(\frac{2}{2e-1}\right)^2\ln(\hat{\varepsilon}^{-1})$ for certain $\varepsilon,\hat{\varepsilon}>0$, and let $g(x,y)=\sqrt{2x\ln(y^{-1})}$. Now:

1. When $test_1$ and $test_2$ are fulfilled, we have that $\gamma=\epsilon+\varepsilon+\hat{\varepsilon}$, $\Delta=g(x,\varepsilon^4/16)$ and $\hat{\Delta}=g(x,\hat{\varepsilon}^{3/2})$.
2. When $test_1$ and $test_3$ are fulfilled (and $test_2$ is not fulfilled), we have that $\gamma=\epsilon+\varepsilon+\hat{\varepsilon}$, $\Delta=g(x,\varepsilon^4/16)$ and $\hat{\Delta}=g(x,\hat{\varepsilon}^2)$.
3. When $test_1$ is fulfilled and $test_3$ is not fulfilled, we have that $\gamma=\epsilon+\varepsilon+\hat{\varepsilon}$, $\Delta=g(x,\varepsilon^4/16)$ and $\hat{\Delta}=\sqrt{(N/2)\ln(1/\varepsilon)}$.
4. When $test_1$ is not fulfilled and $test_2$ is fulfilled, we have that $\gamma=\epsilon+\varepsilon+\hat{\varepsilon}$, $\Delta=\sqrt{(N/2)\ln(1/\varepsilon)}$ and $\hat{\Delta}=g(x,\hat{\varepsilon}^{3/2})$.
5. When $test_1$ and $test_2$ are not fulfilled, and $test_3$ is fulfilled, we have that $\gamma=\epsilon+\varepsilon+\hat{\varepsilon}$, $\Delta=\sqrt{(N/2)\ln(1/\varepsilon)}$ and $\hat{\Delta}=g(x,\hat{\varepsilon}^2)$.
6. When $test_1$, $test_2$ and $test_3$ are not fulfilled, we have that $\gamma=\varepsilon+\hat{\varepsilon}$, $\Delta=\hat{\Delta}=\sqrt{(N/2)\ln(1/\varepsilon)}$.

Lemma 5. Gaussian analysis with the central limit theorem.

Let $X_1, X_2, \ldots, X_N$ be a set of independent and identically distributed Bernoulli random variables that satisfy $\Pr(X_i=1)=p$, and let $X:=\sum_{i=1}^N X_i$. The expected value and variance of X are denoted as $\mu_x:=E[X]$ and

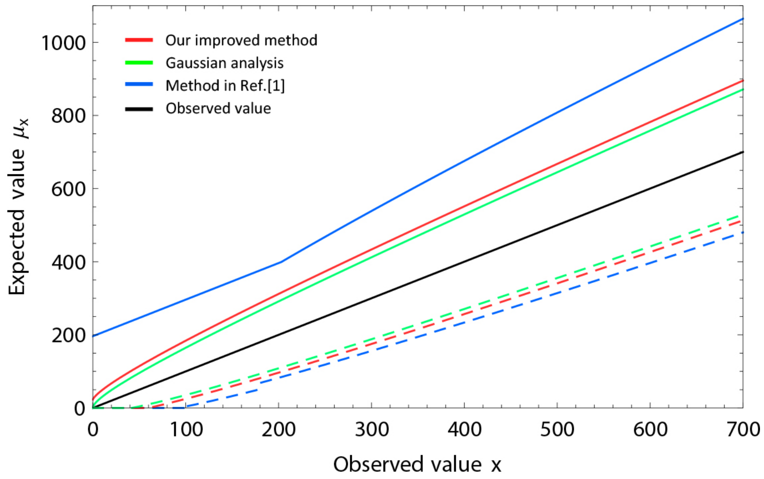

FIG. 1: Expected value as function of the observed value. The colour solid lines represent the upper bound of the expected value, given the failure probability $\epsilon = 10^{-10}$. The colour dotted lines represent the lower bound of the expected value, given the failure probability $\epsilon = 10^{-10}$. The black solid line represents the observed value. The results of our improved method are always inferior but comparable to the Gaussian analysis.

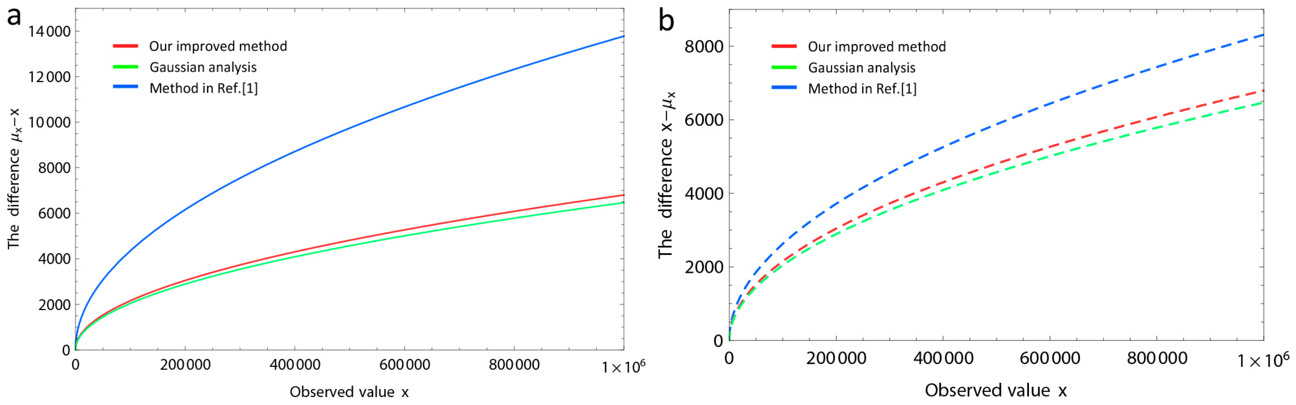

FIG. 2: The difference between the expected and observed values as function of the observed value. **a**, The difference between the upper bound of the expected value and observed value. **b**, The difference between the observed value and the lower bound of the expected value. The failure probability $\epsilon = 10^{-10}$. The results of our improved method are always inferior but comparable to the Gaussian analysis, which means that our rigorous method closes the gap between the rigorous large deviation method in Ref. [1] and the not-sufficiently-rigorous Gaussian analysis.

$\sigma^2 := Var[X]$. An observed outcome of X is represented as x. When $N \to \infty$, $\frac{x-\mu_x}{\sigma}$ approaches a standard normal distribution $N(0,1)$. Thus, as $N \to \infty$, $\sigma = \sqrt{x}$, for any fixed $\beta > 0$ we have

$$\begin{aligned} \Pr[x > \mu_x + \beta\sqrt{x}] &\to \frac{1}{\sqrt{2\pi}}\int_{\beta}^{\infty} e^{-\frac{t^2}{2}} dt = \frac{1}{2}\mathrm{erfc}(\beta/\sqrt{2}), \\ \Pr[x < \mu_x - \beta\sqrt{x}] &\to \frac{1}{\sqrt{2\pi}}\int_{-\infty}^{-\beta} e^{-\frac{t^2}{2}} dt = \frac{1}{2}\mathrm{erfc}(\beta/\sqrt{2}), \end{aligned} \tag{36}$$

where $\mathrm{erfc}(x) = 1 - \frac{2}{\sqrt{\pi}}\int_0^x e^{-t^2} dt$ is the complementary error function.

The Gaussian analysis requires infinite number of independent and identically distributed Bernoulli random variables. Therefore, any rigorous method with finite number of independent (not necessarily identically distributed) Bernoulli random variables should not be better than Gaussian analysis. Without loss of generality, we set each failure probability $\epsilon = \hat{\epsilon} = \varepsilon = \hat{\varepsilon} = 10^{-10}$. Thereby, the three conditions of Lemma 4 [1] become: $test_1$, $\mu_{\mathrm{L}} \geq 84.33$; $test_2$, $\mu_{\mathrm{L}} > 69.08$; $test_3$, $\mu_{\mathrm{L}} > 4.68$. Note that we should have the lower bound $\mu_x = x - \Delta \geq \mu_{\mathrm{L}}$ in Lemma 4. The

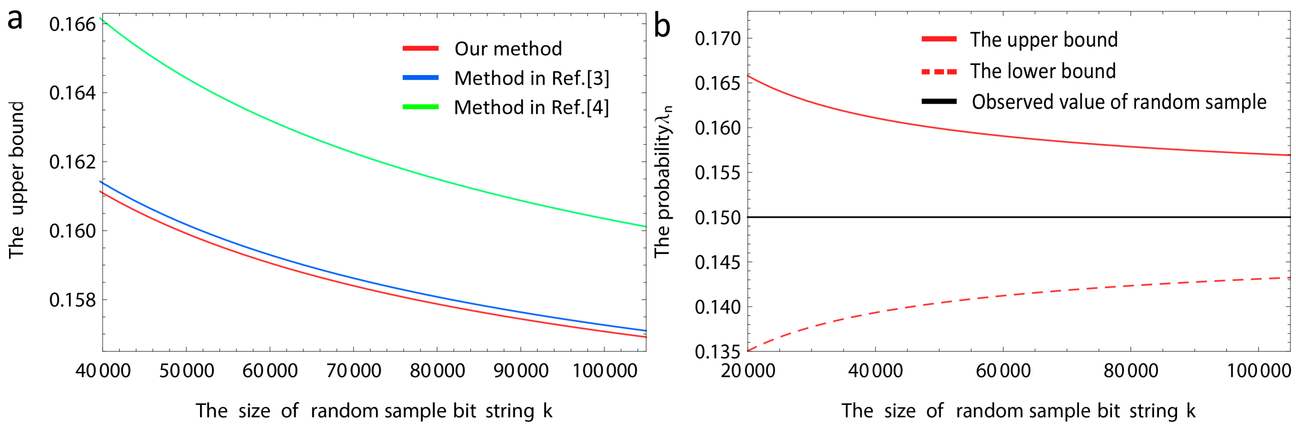

FIG. 3: Comparing the methods of random sampling without replacement. **a**, The upper bound probability of bit value 1 observed in remaining bit string, given $n = 10^6$, $\lambda_k = 0.15$ and $\epsilon = 10^{-10}$. **b**, The upper and lower bound probabilities of bit value 1 observed in remaining bit string in our improved method, given $n = 10^6$, $\lambda_k = 0.15$ and $\epsilon = 10^{-10}$.

three conditions of Lemma 4 further become: $test_1$, $x \geq 203$; $test_2$, $x \geq 181$; $test_3$, $x \geq 102$. For the quantum key distribution system, the probability $\Pr(X_i = 1) = p_i$ is usually very small, which means $x \ll \sqrt{(N/2)\ln(1/\epsilon)}$ and $\Delta = \hat{\Delta} = \sqrt{(N/2)\ln(1/\epsilon)}$ do not apply. Therefore, we can restate Lemma 4 as: if $x \geq 203$, the lower bound of the expected value $\mu_x = x - \sqrt{2x\ln(\epsilon^{-3/2})}$ and the upper bound of the expected value $\mu_x = x + \sqrt{2x\ln(16\epsilon^{-4})}$; if $181 \leq x < 203$, the lower bound of the expected value $\mu_x = x - \sqrt{2x\ln(\epsilon^{-3/2})}$ and the upper bound of the expected value $\mu_x = x + \sqrt{2 \times 203\ln(16\epsilon^{-4})}$; if $102 \leq x < 181$, the lower bound of the expected value $\mu_x = x - \sqrt{2x\ln(\epsilon^{-2})}$ and the upper bound of the expected value $\mu_x = x + \sqrt{2 \times 203\ln(16\epsilon^{-4})}$; if $x < 102$, the lower bound of the expected value $\mu_x = 0$ and the upper bound of the expected value $\mu_x = x + \sqrt{2 \times 203\ln(16\epsilon^{-4})}$. Note that $\epsilon = 10^{-10}$ and we exploit the fact that Δ is the monotonic increasing function of x given fixed failure probability ϵ.

Figures 1 and 2 compare the results among our improved method, the large deviation method in Ref. [1], and the Gaussian analysis. The lower bound of the expected value in Gaussian analysis is always $\mu_x = 0$, given the observed value $x \leq 41$. The upper bound of the expected value in Gaussian analysis is $\mu_x = 0$, given the observed value $x = 0$. The lower bound of the expected value in our improved method is always $\mu_x = 0$, given the observed value $x \leq 59$. The upper bound of the expected value in our improved method is $\mu_x = \ln\epsilon^{-1} = 23.0259$, given the observed value $x = 0$. The lower bound of the expected value in the large deviation method in Ref. [1] is always $\mu_x = 0$, given the observed value $x \leq 101$. The upper bound of the expected value in the large deviation method in Ref. [1] is $\mu_x = \sqrt{406\ln(16\epsilon^{-4})} = 196.264$, given the observed value $x = 0$. The results of our improved method are always inferior but comparable to the Gaussian analysis, which means that our rigorous method closes the gap between the rigorous large deviation method in Ref. [1] and the not-sufficiently-rigorous Gaussian analysis.

Second, we consider the statistical fluctuation of random sampling without replacement. The problem of random sampling without replacement is usually solved by the Serfling inequality [2]. However, the Serfling inequality cannot give very good bound here since this result does not consider the properties of the priori distribution. By using the hypergeometric function distribution, one can provide a good bound even in a high-loss regime [3, 4].

Lemma 6. The upper bound tail inequality of random sampling without replacement [3, 4].

Let $\mathcal{X}_{n+k} := \{x_1, x_2, \cdots, x_{n+k}\}$ be a string of binary bits with $n + k$ size, in which the number of bit value 1 is unknown. Let $\mathcal{X}_k$ be a random sample (without replacement) bit string with k size from $\mathcal{X}_{n+k}$. Let λ_k be the probability of bit value 1 observed in $\mathcal{X}_k$. Let $\mathcal{X}_n$ be the remaining bit string, where the probability of bit value 1 observed in $\mathcal{X}_n$ is λ_n. For any $\epsilon > 0$, we have the upper tail

$$\Pr[\lambda_n \geq \lambda_k + \gamma(n, k, \lambda_k, \epsilon)] \leq \epsilon, \tag{37}$$

where $\gamma(a, b, c, d)$ is the positive root of the following equation [3]

$$h\left[c + \frac{a}{a+b}\gamma(a,b,c,d)\right] - \frac{b}{a+b}h[c] - \frac{a}{a+b}h[c+\gamma(a,b,c,d)] - \frac{1}{2(a+b)}\log_2\frac{a+b}{abc(1-c)d^2} = 0, \tag{38}$$

where $h[x] = -x\log_2 x - (1-x)\log_2(1-x)$ is the Shannon entropy function. By exploiting the Taylor expansion, the

above result can be written as an approximate analytical formula [4],

$$\gamma(a,b,c,d)] = \sqrt{\frac{(a+b)c(1-c)}{ab\ln 2}\log_2\frac{a+b}{abc(1-c)d^2}}. \tag{39}$$

Note that the approximate analytical formula is only true for appropriate parameters a and b, which means that the result of approximate analytical formula Eq. (39) is larger than Eq. (38). The approximate analytical formula is not true, given small a and b.

Figure 3 compares the results among our improved method, the methods in Ref. [3] and Ref. [4] for the random sampling without replacement. The probability of bit vale 1 observed in random sample bit string is $\lambda_k = 0.15$. The size of remaining bit strings is $n = 10^6$. Our bound is the tightest because we avoid excessive inequality scaling. Furthermore, we provide the lower bound tail inequality for random sampling without replacement, which is shown in Fig. 3b.

Supplementary Note 4: Decoy-state analysis with three-intensity

Here, we exploit the decoy-state method with three-intensity ($0 < \omega < \nu$) [5, 6] to estimate the upper bound of the expected yield $\overline{Y}^*_{n,m}$. The upper bound of the expected yield $\overline{Y}^*_{0,0} = \overline{Q}^*_{0,0}$. The upper bound of the expected yield $\overline{Y}^*_{1,1}$, $\overline{Y}^*_{0,2}$ and $\overline{Y}^*_{2,0}$ can be given by

$$\begin{aligned}
\overline{Y}^*_{1,1} &= \frac{e^{2\omega}\overline{Q}^*_{\omega,\omega} - e^{\omega}(\underline{Q}^*_{\omega,0} + \underline{Q}^*_{0,\omega}) + \overline{Q}^*_{0,0}}{\omega^2},\\
\overline{Y}^*_{0,2} &= \frac{\omega e^{\nu}\overline{Q}^*_{0,\nu} - \nu e^{\omega}\underline{Q}^*_{0,\omega} + (\nu-\omega)\overline{Q}^*_{0,0}}{\nu\omega(\nu-\omega)/2},\\
\overline{Y}^*_{2,0} &= \frac{\omega e^{\nu}\overline{Q}^*_{\nu,0} - \nu e^{\omega}\underline{Q}^*_{\omega,0} + (\nu-\omega)\overline{Q}^*_{0,0}}{\nu\omega(\nu-\omega)/2}.
\end{aligned} \tag{40}$$

The upper bound of the expected yield $\overline{Y}^*_{0,4}$ and $\overline{Y}^*_{4,0}$ can be given by

$$\begin{aligned}
\overline{Y}^*_{0,4} &= \min\left\{1,\ \frac{\omega e^{\nu}\overline{Q}^*_{0,\nu} - \nu e^{\omega}\underline{Q}^*_{0,\omega} + (\nu-\omega)\overline{Q}^*_{0,0}}{\nu\omega(\nu^3-\omega^3)/4!}\right\},\\
\overline{Y}^*_{4,0} &= \min\left\{1,\ \frac{\omega e^{\nu}\overline{Q}^*_{\nu,0} - \nu e^{\omega}\underline{Q}^*_{\omega,0} + (\nu-\omega)\overline{Q}^*_{0,0}}{\nu\omega(\nu^3-\omega^3)/4!}\right\}.
\end{aligned} \tag{41}$$

The upper bound of the expected yield $\overline{Y}^*_{1,3}$ and $\overline{Y}^*_{3,1}$ can be given by

$$\begin{aligned}
\overline{Y}^*_{1,3} &= \min\left\{1,\frac{(\omega e^{\nu+\omega}\overline{Q}^*_{\omega,\nu} + (\nu-\omega)e^{\omega}\overline{Q}^*_{\omega,0} + \nu e^{\omega}\overline{Q}^*_{0,\omega}) - (\omega e^{\nu}\underline{Q}^*_{0,\nu} + \nu e^{2\omega}\underline{Q}^*_{\omega,\omega} + (\nu-\omega)\underline{Q}^*_{0,0})}{\nu\omega^2(\nu^2-\omega^2)/3!}\right\}\\
\overline{Y}^*_{3,1} &= \min\left\{1,\frac{(\omega e^{\nu+\omega}\overline{Q}^*_{\nu,\omega} + \nu e^{\omega}\overline{Q}^*_{\omega,0} + (\nu-\omega)e^{\omega}\overline{Q}^*_{0,\omega}) - (\omega e^{\nu}\underline{Q}^*_{\nu,0} + \nu e^{2\omega}\underline{Q}^*_{\omega,\omega} + (\nu-\omega)\underline{Q}^*_{0,0})}{\nu\omega^2(\nu^2-\omega^2)/3!}\right\}
\end{aligned} \tag{42}$$

The upper bound of the expected yield $\overline{Y}^*_{2,2}$ can be given by

$$\begin{aligned}
\overline{Y}^*_{2,2} = \min\Big\{1, \frac{1}{\nu^2\omega^2(\nu-\omega)^2/4}&[\omega^2 e^{2\nu}\overline{Q}^*_{\nu,\nu} + \nu^2 e^{2\omega}\overline{Q}^*_{\omega,\omega} + \omega(\nu-\omega)e^{\nu}(\overline{Q}^*_{\nu,0} + \overline{Q}^*_{0,\nu}) + (\nu-\omega)^2\overline{Q}^*_{0,0}]\\
&-[\nu\omega e^{\nu+\omega}(\underline{Q}^*_{\nu,\omega} + \underline{Q}^*_{\omega,\nu}) + \nu(\nu-\omega)e^{\omega}(\underline{Q}^*_{\omega,0} + \underline{Q}^*_{0,\omega})]\Big\}.
\end{aligned} \tag{43}$$

Let $\overline{s}^*_{n,m} = \overline{Y}^*_{n,m}N_{\rm X}\sum_{a,b}p_ap_bP^a_nP^b_m$ be upper bound of the expected bit number in the X basis given that Alice and Bob send n-photon and m-photon. By using the upper tail of the multiplicative Chernoff bound in Lemma 2, we can

estimate the upper bound of the observed bit number $\overline{s}_{n,m}$ given by $\overline{s}^{*}_{n,m}$ with failure probability ϵ_2. Thereby, the upper bound of the observed yield $\overline{Y}_{n,m} = \overline{s}_{n,m}/\left(N_{\mathrm{X}}\sum_{a,b}p_a p_b p_n^a p_m^b\right)$. For the case of $n+m\geq 5$, we let the upper bound of the observed yield $\overline{Y}_{n,m}=1$.

In our simulation, we have [6]

$$\begin{aligned} Q_{\mathrm{Z}} &= (1-p_d)[1-(1-2p_d)e^{-2\mu\eta}], \\ E_{\mathrm{Z}} &= [e_{d_{\mathrm{Z}}}Q_{\mathrm{Z}}^{C} + (1-e_{d_{\mathrm{Z}}})Q_{\mathrm{Z}}^{E}]/Q_{\mathrm{Z}}, \\ Q_{\mathrm{Z}}^{E} &= p_d(1-p_d)e^{-2\mu\eta}, \\ Q_{\mathrm{Z}}^{C} &= (1-p_d)[1-(1-p_d)e^{-2\mu\eta}], \\ Q_{a,b} &= 2(1-p_d)e^{-\frac{1}{2}(a+b)\eta}I_0(\sqrt{ab}\eta) - 2(1-p_d)^2e^{-(a+b)\eta}. \end{aligned} \tag{44}$$

where $I_0(x)$ is the modified Bessel function of the first kind, p_d is the dark count rate, $e_{d_{\mathrm{Z}}}$ is the misalignment rate of the Z basis, η is the overall efficiency between Alice (Bob) and Charlie.

[1] Curty, M. *et al.* Finite-key analysis for measurement-device-independent quantum key distribution. *Nature Commun.* **5**, 3732 (2014).
[2] Tomamichel, M., Lim, C. C. W., Gisin, N. & Renner, R. Tight finite-key analysis for quantum cryptography. *Nature Commun.* **3**, 634 (2012).
[3] Fung, C.-H. F., Ma, X. & Chau, H. F. Practical issues in quantum-key-distribution postprocessing. *Phys. Rev. A* **81**, 012318 (2010).
[4] Lim, C. C. W., Curty, M., Walenta, N., Xu, F. & Zbinden, H. Concise security bounds for practical decoy-state quantum key distribution. *Phys. Rev. A* **89**, 022307 (2014).
[5] Grasselli, F. & Curty, M. Practical decoy-state method for twin-field quantum key distribution. *arXiv:1902.10034* (2019).
[6] Yin, H.-L. & Chen, Z.-B. Coherent-state-based twin-field quantum key distribution. *arXiv:1901.05009* (2019).
